# Comparison of Real-Time Polymerase Chain Reaction and Anaerobic Culture for Detecting and Quantifying Porphyromonas gingivalis in Subgingival Plaque From Periodontitis and Healthy Subjects

**DOI:** 10.7759/cureus.101778

**Published:** 2026-01-18

**Authors:** T Rachita, Shubha Kumaraswamy, Sravya Kodati, Thouseef Ansari, Shivani Karre, Amelia David, Rahul Tiwari, Seema Gupta

**Affiliations:** 1 Department of Periodontology, Index Institute of Dental Sciences, Indore, IND; 2 Department of Oral Pathology, Sri Siddhartha Dental College, Tumkur, IND; 3 Department of Oral Pathology, Government Dental College and Hospital, Vijayawada, IND; 4 Department of Periodontology, Azeezia Dental College, Kollam, IND; 5 Division of General Dentistry, Loma Linda University School of Dentistry, Loma Linda, USA; 6 Department of Periodontology, Loma Linda University School of Dentistry, Loma Linda, USA; 7 Department of Research Cell, Dr. D. Y. Patil Dental College and Hospital, Dr. D. Y. Patil Vidyapeeth (Deemed to be University), Pune, IND; 8 Department of Orthodontics, Kothiwal Dental College and Research Centre, Moradabad, IND

**Keywords:** anaerobic, culture, periodontitis, porphyromonas gingivalis, real-time polymerase chain reaction

## Abstract

Background

*Porphyromonas gingivalis* (*P. gingivalis*) is a key pathogen in chronic periodontitis that drives dysbiosis and tissue destruction. Anaerobic culture often underestimates fastidious bacteria, whereas real-time polymerase chain reaction (RT-PCR) can provide higher sensitivity and accurate quantification. This study compared both methods for detecting and quantifying *P. gingivalis* in subgingival plaque from patients with chronic periodontitis and healthy controls.

Methodology

In total, 32 participants (16 with chronic periodontitis and 16 healthy) were included. Pooled subgingival plaque samples were analyzed by anaerobic culture on selective media and by RT-PCR targeting the 16S rRNA gene with a TaqMan probe and standard curve quantification. Clinical parameters were recorded (probing depth (PD), clinical attachment loss (CAL), and gingival index (GI)), and data were analyzed using t-tests, chi-square tests, Pearson’s correlation, and diagnostic metrics.

Results

Participants with chronic periodontitis had significantly higher clinical parameters (PD = 6.38 ± 0.96 mm, CAL = 3.63 ± 1.09 mm, GI = 2.50 ± 0.22; all p = 0.001) and *P. gingivalis* loads (culture = 82.13 ± 54.26 CFU/mL, p = 0.031; RT-PCR = 97.44 ± 59.02 CFU equivalents/mL, p = 0.039) than controls. Bacterial load showed moderate positive correlations with clinical parameters (r = 0.35-0.42, p < 0.05). RT-PCR detected *P. gingivalis* in 59.4% of samples versus 50% by culture, achieving 100% sensitivity and 90.6% accuracy.

Conclusions

RT-PCR is more sensitive than culture for *P. gingivalis* detection, supporting its use for improved periodontal diagnosis and risk assessment.

## Introduction

Periodontitis is a long-standing inflammatory condition marked by the gradual breakdown of the supporting structures of the teeth, which can eventually result in tooth loss in adults. The disease is mainly initiated by an imbalance in the subgingival microbial community, where certain pathogenic bacteria are key contributors to its development [1,2]. Among these microorganisms, *Porphyromonas gingivalis* (*P. gingivalis*), a gram-negative, obligate anaerobe classified within the “red complex,” has been widely recognized as a major contributor to the onset and advancement of chronic periodontitis. The pathogenicity of *P. gingivalis* is attributed to a range of virulence determinants, such as fimbriae and lipopolysaccharide, which facilitate tissue penetration, subversion of host immune responses, and disturbance of host homeostasis, thereby establishing its central role in periodontal tissue destruction [3,4].

Accurate identification and quantification of* P. gingivalis* in subgingival plaque are essential for risk assessment, diagnosis, and tailored treatment planning of periodontitis. Traditional anaerobic culture techniques have long been considered the gold standard for bacterial identification, allowing for the characterization of physiological traits and virulence factors. However, culture methods have significant limitations, including the inability to detect non-viable or fastidious organisms, prolonged incubation periods, high costs, and potential underestimation due to strict growth requirements [4,5].

In contrast, molecular techniques, such as real-time polymerase chain reaction (RT-PCR), offer superior sensitivity and specificity by targeting species-specific genetic markers, such as the 16S rRNA gene. RT-PCR enables precise quantification through continuous monitoring of amplification, detection of both viable and non-viable cells, and a reduced risk of contamination compared to conventional PCR [6,7]. Some studies have demonstrated that RT-PCR detects *P. gingivalis* levels more accurately than culture, particularly in subgingival samples [4,8]. However, other studies have reported good agreement between RT-PCR and culture methods for the detection of *P. gingivalis* in plaque samples from patients with periodontitis [9,10].

Despite the growing evidence supporting molecular diagnostics, few studies have directly compared RT-PCR and culture techniques for* P. gingivalis *detection in periodontally healthy and chronic periodontitis patients within similar populations. Therefore, this study aimed to detect and quantify *P. gingivalis* in subgingival plaque samples from periodontally healthy individuals and patients with chronic periodontitis using RT-PCR and anaerobic culture techniques. The specific objectives were as follows: (1) to assess the prevalence and quantity of *P. gingivalis* in both groups using each method, (2) to compare the sensitivity and accuracy of RT-PCR versus culture in identification and quantification, and (3) to correlate the bacterial load with clinical parameters of periodontal disease.

## Materials and methods

Study design

This observational microbiological study was conducted over a period of one year at the Department of Periodontology as a thesis project at Sri Rajiv Gandhi College of Dental Sciences and Hospital, Bangalore, Karnataka, India. Ethical approval was obtained from the Institutional Ethics Committee (approval number: SRGCDS/2021/143), and all participants provided written informed consent.

A total of 32 participants were recruited and divided into the following two groups: a test group consisting of 16 participants with chronic periodontitis and a control group of 16 periodontally healthy participants. The sample size was determined a priori using G*Power software (version 3.1.9.2, Heinrich Heine University, Düsseldorf, Germany). Based on an expected correlation coefficient of 0.4 between P. gingivalis load and periodontal probing depth (PD) [11], a significance level (α) of 0.05, and a statistical power of 80%, a minimum of 32 participants was required for this study. These participants were then recruited according to the study’s inclusion criteria and were allocated to the control and test groups.

Participants and inclusion/exclusion criteria

Participants were selected from patients aged between 25 and 50 years, including both men and women. For the control group (periodontally healthy subjects), the inclusion criteria were PD ≤3 mm and a gingival index (GI) ≤1, with no signs of inflammation or bleeding on probing [12]. For the test group (chronic periodontitis patients), the inclusion criteria were PD >3 mm, GI ≥2, presence of inflammation, bleeding on probing, and clinical attachment loss (CAL) ≥2 mm. The exclusion criteria applied to both groups included subjects who had received periodontal treatment or antibiotics in the past six months, those with known systemic diseases, smokers, tobacco users, and pregnant or lactating women. Recruitment ensured a balanced distribution of age and sex to minimize bias.

Clinical assessment

Clinical parameters were evaluated using UNC-15 periodontal probes (Hu-Friedy, Chicago, IL, USA). PD was recorded as the distance from the gingival margin to the base of the periodontal pocket, while clinical attachment level was determined by measuring the distance from the cementoenamel junction to the base of the pocket. Measurements were taken at six sites per tooth, excluding third molars, by a calibrated examiner, with intra-examiner reliability assessed using kappa statistics (>0.85). All assessments were performed before sample collection to categorize the patients and correlate with the microbiological findings.

Sample collection

Subgingival plaque samples were collected from the deepest periodontal pocket in each quadrant using sterile Gracey curettes (Hu-Friedy, Chicago, IL, USA). Supragingival plaque was first removed using sterile cotton pellets, and the sites were isolated using cotton rolls to prevent contamination. The plaque collected from the four sites was pooled into 1.5 mL of reduced transport fluid (RTF) prepared in-house (composition: 0.045% K_2_HPO_4_, 0.09% NaCl, 0.09% (NH_4_)_2_SO_4_, 0.018% EDTA, 0.04% Na_2_CO_3_, 0.09% dithiothreitol, pH 8.0). Samples were transported immediately to the laboratory and processed for culture within 48 hours or stored at -80°C for RT-PCR analysis.

Anaerobic culture technique

Samples in RTF were vortexed for 30 seconds and serially diluted in sterile phosphate-buffered saline. Aliquots (0.1 mL) were plated on tryptic soy agar with 5% sheep blood (BD BBL™ Prepared Plated Media, Becton, Dickinson and Company, Franklin Lakes, NJ, USA) supplemented with hemin (5 μg/mL) and menadione (1 μg/ml). Plates were incubated anaerobically at 37°C for 7-10 days in an anaerobic jar using the anaerobe container system (GasPak™ EZ, Becton, Dickinson and Company, Franklin Lakes, NJ, USA) [13].

Colonies of *P. gingivalis* were identified based on their distinctive black pigmentation, Gram staining characteristics (Gram-negative rods), obligate anaerobic growth, inability to ferment glucose, indole positivity, and positive hemagglutination with 3% sheep erythrocytes. The reference strain *P. gingivalis* ATCC 33277 (American Type Culture Collection, Manassas, VA, USA) served as the comparator in this study. Colony-forming units (CFUs) were enumerated manually, and bacterial counts were expressed as CFU per sample [4].

DNA extraction and RT-PCR technique

Genomic DNA was isolated from subgingival plaque samples using the QIAamp DNA Mini Kit (QIAGEN, Hilden, Germany) following the manufacturer’s instructions, with an initial boiling step at 100°C for 10 minutes to ensure cell lysis. The concentration and purity of the extracted DNA were assessed using a NanoDrop™ 2000 spectrophotometer (Thermo Fisher Scientific, Waltham, MA, USA), and samples were stored at −20°C until further analysis. RT-PCR was conducted to amplify the 16S rRNA gene of *P. gingivalis* using an RT-PCR detection system (CFX96, Bio-Rad Laboratories, Hercules, CA, USA). Each 25 µL reaction mixture comprised 12.5 µL of TaqMan Universal PCR Master Mix (Thermo Fisher Scientific, Waltham, MA, USA), 300 nM of each primer, 200 nM of a fluorescently labeled probe, and 5 µL of template DNA. The primers were custom-synthesized (Sigma-Aldrich, St. Louis, MO, USA), with the forward primer sequence 5′-AGG CAG CTT GCC ATA CTG CG-3′ and the reverse primer sequence 5′-ACT GTT AGC AAC TAC CGA TGT-3′. A TaqMan probe specific to the *P. gingivalis* 16S rRNA gene (5′-FAM-CACTGAACTCAAGCCCGGCAGTTTCAA-TAMRA-3′) was used for detection [4,14].

The thermal cycling conditions consisted of an initial denaturation step at 95°C for 10 minutes, followed by 40 amplification cycles of denaturation at 95°C for 15 seconds and annealing/extension at 60°C for 1 minute. A standard curve was established using serial dilutions of DNA obtained from *P. gingivalis* ATCC 33277 (American Type Culture Collection, Manassas, VA, USA), and nuclease-free water was included as a negative control in each run. Quantitative analysis was performed based on cycle threshold (Ct) values, and bacterial levels were expressed as genome equivalents per sample.

Statistical analysis

Data were analyzed using SPSS software (version 20.0; IBM Corporation, Armonk, NY, USA). The normality of the quantitative data distribution was assessed and confirmed using the Shapiro-Wilk test. Categorical demographic variables (age group and sex) were compared between the control and test groups using the chi-square test. Independent samples t-tests were used to compare the means of continuous clinical parameters (PD, CAL, GI) and the quantitative load of P. gingivalis (both culture and RT-PCR) between the groups. Pearson’s correlation coefficient was calculated to determine the strength of the association between bacterial load and clinical parameters. The diagnostic performance metrics of RT-PCR versus culture were calculated, with culture as the reference standard.

## Results

The study groups were comparable in terms of age (p = 0.476) and sex distribution (p = 0.287), with no significant differences (Table 1). As expected, patients with chronic periodontitis (test group) exhibited significantly higher PD (6.38 ± 0.96 mm vs. 2.38 ± 0.50 mm), CAL (3.63 ± 1.09 mm vs. 0.12 ± 0.08 mm), and GI (2.50 ± 0.22 vs. 0.49 ± 0.20) than healthy controls (all p = 0.001). *P. gingivalis* loads were significantly higher in the test group than in controls, both by anaerobic culture (82.13 ± 54.26 CFU/sample vs. 46.81 ± 31.42 CFU/sample; p = 0.031) and RT-PCR (97.44 ± 59.02 CFU equivalents vs. 57.81 ± 44.21 CFU equivalents; p = 0.039) (Table 1).

**Table 1:** Demographic and clinical characteristics of the study groups and quantification of Porphyromonas gingivalis (P. gingivalis) by culture and RT-PCR. Control group: periodontally healthy subjects; Test group: chronic periodontitis patients. Data are presented as frequency (%) for categorical variables and mean ± standard deviation for continuous variables. Categorical variables compared using the chi-square test; continuous variables using the independent samples t-test. *: p < 0.05 indicates statistical significance. CFU = colony-forming unit; RT-PCR = real-time polymerase chain reaction

Parameters	Category	Control (n = 16)	Test (n = 16)	Test value	P-value
Age groups	<30 years	6 (37.5%)	8 (50%)	0.50	0.476
	>30 years	10 (62.5%)	8 (50%)		
Sex	Male	9 (56.25%)	6 (37.5%)	1.12	0.287
	Female	7 (43.75%)	10 (62.5%)		
Pocket depth (mm)	Mean ± SD	2.38 ± 0.50	6.38 ± 0.96	14.78	0.001*
Clinical attachment loss (mm)	Mean ± SD	0.12 ± 0.08	3.63 ± 1.09	12.84	0.001*
Gingival index	Mean ± SD	0.49 ± 0.20	2.50 ± 0.22	27.04	0.001*
*P. gingivalis* (culture)	CFU/sample	46.81 ± 31.42	82.13 ± 54.26	2.25	0.031*
*P. gingivalis* (RT-PCR)	CFU equivalents	57.81 ± 44.21	97.44 ± 59.02	2.14	0.039*

The quantity of *P. gingivalis *determined by both methods showed similar moderate positive correlations with periodontal clinical parameters in all participants (Table 2). The strongest associations were with PD (r ≈ 0.41-0.42), followed by CAL (r ≈ 0.38) and GI (r ≈ 0.35-0.36), all of which were statistically significant.

**Table 2:** Pearson correlation coefficients between clinical parameters and Porphyromonas gingivalis loads determined by culture and RT-PCR (n = 32). *: p < 0.05 indicates statistical significance. RT-PCR = real-time polymerase chain reaction

Outcome variables		*P. gingivalis *(culture method)	*P. gingivalis* (RT-PCR method)
Pocket depth (mm)	Pearson correlation coefficient (r)	0.409	0.422
	P-value	0.02*	0.02*
Clinical attachment loss (mm)	Pearson correlation coefficient (r)	0.378	0.385
	P-value	0.03*	0.03*
Gingival index	Pearson correlation coefficient (r)	0.350	0.362
	P-value	0.04*	0.04*

RT-PCR demonstrated higher detection rates of *P. gingivalis* than anaerobic culture (Table 3). Notably, in patients with chronic periodontitis, RT-PCR identified the pathogen in 100% of samples, including both culture-negative cases, whereas culture was positive in 87.5% of cases. In healthy controls, RT-PCR detected one additional positive sample (18.75% vs. 12.5%).

**Table 3:** Comparison of detection methods for Porphyromonas gingivalis in subgingival plaque samples from periodontally healthy (Control) and chronic periodontitis (Test) participants using anaerobic culture as the reference standard. Diagnostic metrics were calculated using anaerobic culture as the reference standard. Values are n (%). Sensitivity = TP/(TP+FN); specificity = TN/(TN+FP); PPV = TP/(TP+FP); NPV = TN/(TN+FN). TP = true positive; FN = false negative; FP = false positive; TN = true negative; RT-PCR = real-time polymerase chain reaction

Participant group	Culture result	RT-PCR positive	RT-PCR negative	Total
Control as periodontally healthy participants (n = 16)	Positive	2 (12.50%)	0 (0.00%)	2 (12.50%)
	Negative	1 (6.25%)	13 (81.25%)	14 (87.50%)
	Total	3 (18.75%)	13 (81.25%)	16 (100.00%)
Test as participants with chronic periodontitis (n = 16)	Positive	14 (87.50%)	0 (0.00%)	14 (87.50%)
	Negative	2 (12.50%)	0 (0.00%)	2 (12.50%)
	Total	16 (50.00%)	0 (0.00%)	16 (100.00%)
Overall (n = 32)	Positive	16 (50.00%)	0 (0.00%)	16 (50.00%)
	Negative	3 (9.37%)	13 (40.63%)	16 (50.00%)
	Total	19 (59.37%)	13 (40.63%)	32 (100.00%)

When evaluated against culture as the reference standard, RT-PCR exhibited perfect sensitivity (100%) overall and in each group, indicating no false-negative results (Table 4). Specificity was high in the controls (92.9%) but zero in the test group owing to the two culture-negative/RT-PCR-positive discrepancies. The overall accuracy was 90.6%, with positive and negative predictive values of 84.2% and 100%, respectively.

**Table 4:** Diagnostic performance of RT-PCR compared to anaerobic culture for the detection of Porphyromonas gingivalis in subgingival plaque samples. *: Not calculable due to the absence of PCR-negative results in culture-negative test samples. Anaerobic culture was used as the reference standard. RT-PCR = real-time polymerase chain reaction; CI = confidence interval; PPV = positive predictive value; NPV = negative predictive value

Group	Sensitivity (95% CI)	Specificity (95% CI)	PPV (95% CI)	NPV (95% CI)	Accuracy (95% CI)
Control (periodontally healthy)	100% (34.2%–100%)	92.9% (68.5%–99.6%)	66.7% (20.8%–94.2%)	100% (77.0%–100%)	93.8% (71.7%–99.7%)
Test (chronic periodontitis)	100% (78.5%–100%)	0% (0%–70.1%)	87.5% (62.5%–97.7%)	Not calculable*	87.5% (63.9%–97.8%)
Overall	100% (80.6%–100%)	81.3% (55.3%–94.6%)	84.2% (61.4%–95.2%)	100% (77.0%–100%)	90.6% (75.8%–97.3%)

## Discussion

The present study revealed a markedly higher prevalence and load of *P. gingivalis* in subgingival plaque samples from patients with chronic periodontitis than in periodontally healthy individuals, as detected by both anaerobic culture and RT-PCR. This qualitative difference underscores the established role of pathogens in periodontal disease progression, aligning with their classification within the red complex of periodontal pathogens. Previous investigations have consistently demonstrated elevated levels of this bacterium in diseased sites relative to healthy sites, attributing the disparity to its virulence factors that promote dysbiosis and tissue destruction. These observed patterns reinforce the notion that *P. gingivalis *abundance correlates with disease severity, although the exact threshold for pathogenicity remains context-dependent [3,15,16].

When comparing detection methods, RT-PCR exhibited superior sensitivity to anaerobic culture, identifying the pathogen in a greater proportion of samples across both groups. This finding concurs with multiple prior studies that reported RT-PCR as being more effective for quantifying *P. gingivalis*, particularly in subgingival plaque from periodontitis cohorts [4,8]. For instance, earlier work highlighted RT-PCR’s ability to detect low-abundance or non-culturable cells, leading to higher positivity rates than culture-based approaches [4]. In contrast, some studies have shown good concordance between the two techniques, especially when culture conditions are optimized for fastidious anaerobes [9,10]. The discrepancies in our results, where RT-PCR identified additional positives in culture-negative samples from the periodontitis group, may stem from the limitations of culture in recovering viable cells under stringent anaerobic requirements or from overgrowth by competing flora. RT-PCR, by targeting the 16S rRNA gene, captures both viable and non-viable DNA, potentially inflating detection in chronic infections in which bacterial remnants persist. This methodological advantage explains the perfect sensitivity of RT-PCR relative to culture as the reference, although specificity varied by group, reflecting the influence of disease status on bacterial viability [17].

The reasons for these methodological differences are multifaceted. Anaerobic culture, while allowing phenotypic characterization, is labor-intensive and prone to underestimation due to the slow growth of bacteria and their sensitivity to oxygen exposure during sampling and transport. RT-PCR mitigates these issues through rapid amplification and quantification using standard curves, offering reproducibility and reduced contamination risk [18]. However, variations across studies could arise from primer specificity. We used Ashimoto-derived primers and a TaqMan probe targeting a conserved 16S rRNA region, similar to the protocols used in comparable studies [4]; however, probe design differences may affect amplification efficiency. Additionally, sample pooling from multiple sites in our methodology may have diluted low-level detections in healthy participants, contrasting with site-specific sampling in some prior comparisons that reported even greater sensitivity gaps. Population factors, such as geographic or ethnic variations in microbial profiles, also contribute to these differences. Studies in diverse cohorts have noted inconsistent agreement between methods, possibly due to strain heterogeneity or host immune responses affecting the culturability [9,19].

Moderate positive correlations between *P. gingivalis* load and clinical parameters were consistent across both detection methods, suggesting that bacterial quantity reflects disease activity. This mirrors the findings of longitudinal studies linking higher pathogen burdens to progressive tissue breakdown, emphasizing the keystone role of *P. gingivalis* in dysbiotic shifts [4,18]. Differences in correlation strength compared to previous reports may be related to our focus on chronic rather than aggressive periodontitis, where associations are often stronger due to rapid progression. Moreover, using CFU equivalents for RT-PCR quantification facilitated direct comparison, although genome copy adjustments could refine these links in future studies.

Clinically, these results advocate for RT-PCR as the preferred diagnostic tool in periodontal practice, enabling faster risk assessment and personalized interventions such as targeted antimicrobials or adjunctive therapies. Its higher sensitivity could improve early detection in at-risk populations, potentially reducing the disease burden through timely management. However, the limitations of this study include the small sample size, which may limit generalizability, and the cross-sectional design, which precludes causal inferences. Reliance on pooled samples might overlook site-specific variations, and the absence of viability assays (such as propidium monoazide treatment) could lead to overestimation of active infections. Future studies should incorporate larger cohorts, longitudinal tracking, and multiplex assays for polymicrobial analyses to address these gaps.

## Conclusions

This study demonstrates that *P. gingivalis* is significantly more prevalent and abundant in patients with chronic periodontitis than in healthy individuals, with loads positively correlated with disease severity. RT-PCR was more sensitive than anaerobic culture, detecting the pathogen in all periodontitis samples and additional cases missed by culture. These findings highlight the superiority of RT-PCR as a diagnostic tool for the accurate identification and quantification of *P. gingivalis*, facilitating better risk assessment and targeted therapy in periodontal practice.

## References

[1] Könönen E, Gursoy M, Gursoy UK (2019). Periodontitis: a multifaceted disease of tooth-supporting tissues. J Clin Med.

[2] Ji S, Choi YS, Choi Y (2015). Bacterial invasion and persistence: critical events in the pathogenesis of periodontitis?. J Periodontal Res.

[3] Nuñez-Belmar J, Morales-Olavarria M, Vicencio E, Vernal R, Cárdenas JP, Cortez C (2022). Contribution of -omics technologies in the study of Porphyromonas gingivalis during periodontitis pathogenesis: a minireview. Int J Mol Sci.

[4] Boutaga K, van Winkelhoff AJ, Vandenbroucke-Grauls CM, Savelkoul PH (2003). Comparison of real-time PCR and culture for detection of Porphyromonas gingivalis in subgingival plaque samples. J Clin Microbiol.

[5] Houpikian P, Raoult D (2002). Traditional and molecular techniques for the study of emerging bacterial diseases: one laboratory's perspective. Emerg Infect Dis.

[6] Bustin SA (2000). Absolute quantification of mRNA using real-time reverse transcription polymerase chain reaction assays. J Mol Endocrinol.

[7] Doungudomdacha S, Rawlinson A, Douglas CW (2000). Enumeration of Porphyromonas gingivalis, Prevotella intermedia and Actinobacillus actinomycetemcomitans in subgingival plaque samples by a quantitative-competitive PCR method. J Med Microbiol.

[8] Riggio MP, Macfarlane TW, Mackenzie D, Lennon A, Smith AJ, Kinane D (1996). Comparison of polymerase chain reaction and culture methods for detection of Actinobacillus actinomycetemcomitans and Porphyromonas gingivalis in subgingival plaque samples. J Periodontal Res.

[9] Jervøe-Storm PM, Koltzscher M, Falk W, Dörfler A, Jepsen S (2005). Comparison of culture and real-time PCR for detection and quantification of five putative periodontopathogenic bacteria in subgingival plaque samples. J Clin Periodontol.

[10] Lau L, Sanz M, Herrera D, Morillo JM, Martín C, Silva A (2004). Quantitative real-time polymerase chain reaction versus culture: a comparison between two methods for the detection and quantification of Actinobacillus actinomycetemcomitans, Porphyromonas gingivalis and Tannerella forsythensis in subgingival plaque samples. J Clin Periodontol.

[11] Kawada M, Yoshida A, Suzuki N, Nakano Y, Saito T, Oho T, Koga T (2004). Prevalence of Porphyromonas gingivalis in relation to periodontal status assessed by real-time PCR. Oral Microbiol Immunol.

[12] Löe H (1967). The gingival index, the plaque index and the retention index systems. J Periodontol.

[13] Balan DK, John J, Ahamed S, Syam N, Sudhakaran G, M L, Ali H (2024). An anaerobic culture study to assess the prevalence of Porphyromonas gingivalis in periodontal disease incidences among adults. Cureus.

[14] Ingalagi P, Bhat KG, Kulkarni RD, Kotrashetti VS, Kumbar V, Kugaji M (2022). Detection and comparison of prevalence of Porphyromonas gingivalis through culture and real time-polymerase chain reaction in subgingival plaque samples of chronic periodontitis and healthy individuals. J Oral Maxillofac Pathol.

[15] Aleksijević LH, Aleksijević M, Škrlec I, Šram M, Šram M, Talapko J (2022). Porphyromonas gingivalis virulence factors and clinical significance in periodontal disease and coronary artery diseases. Pathogens.

[16] How KY, Song KP, Chan KG (2016). Porphyromonas gingivalis: an overview of periodontopathic pathogen below the gum line. Front Microbiol.

[17] Moorlag SJ, Coolen JP, van den Bosch B, Jin EH, Buil JB, Wertheim HF, Melchers WJ (2023). Targeting the 16S rRNA gene by reverse complement PCR next-generation sequencing: specific and sensitive detection and identification of microbes directly in clinical samples. Microbiol Spectr.

[18] Kotsilkov K, Popova C, Boyanova L, Setchanova L, Mitov I (2015). Comparison of culture method and real-time PCR for detection of putative periodontopathogenic bacteria in deep periodontal pockets. Biotechnol Biotechnol Equip.

[19] Wang BY, Burgardt G, Parthasarathy K (2023). Influences of race/ethnicity in periodontal treatment response and bacterial distribution, a cohort pilot study. Front Oral Health.

